# Modes of Action of Biocontrol Agents and Elicitors for sustainable Protection against Bacterial Canker of Tomato

**DOI:** 10.3390/microorganisms11030726

**Published:** 2023-03-11

**Authors:** Salma Benchlih, Qassim Esmaeel, Kamal Aberkani, Abdessalem Tahiri, Zineb Belabess, Rachid Lahlali, Essaid Ait Barka

**Affiliations:** 1 Phytopathology Unit, Department of Plant Protection, Ecole Nationale d’Agriculture de Meknès, Km 10, Rte Haj Kaddour, BP S/40, Meknes 50001, Morocco; 2 Unité de Recherche Résistance Induite et Bio-Protection des Plantes-EA 4707-USC INRAE1488, Université de Reims Champagne-Ardenne, 51100 Reims, France; 3 Faculté Poly-Disciplinaire de Nador, University Mohammed Premier, Oujda 60000, Morocco; 4 Plant Protection Laboratory, Regional Center of Agricultural Research of Meknes, National Institute of Agricultural Research, Km 13, Route Haj Kaddour, BP.578, Meknes 50001, Morocco

**Keywords:** bacterial canker, *C michiganensis* subsp. *michiganensis*, biocontrol, plant growth promoting rhizobacteria, elicitors, sustainable agriculture

## Abstract

Tomato is one of the world’s most commonly grown and consumed vegetables. However, it can be attacked by the Gram-positive bacterium *Clavibacter michiganensis* subsp. *michiganensis* (*Cmm*), which causes bacterial canker on tomato plants, resulting in significant financial losses in field production and greenhouses worldwide. The current management strategies rely principally on the application of various chemical pesticides and antibiotics, which represent a real danger to the environment and human safety. Plant growth-promoting rhizobacteria (PGPR) have emerged as an attractive alternative to agrochemical crop protection methods. PGPR act through several mechanisms to support plant growth and performance, while also preventing pathogen infection. This review highlights the importance of bacterial canker disease and the pathogenicity of *Cmm*. We emphasize the application of PGPR as an ecological and cost-effective approach to the biocontrol of *Cmm*, specifying the complex modes of biocontrol agents (BCAs), and presenting their direct/indirect mechanisms of action that enable them to effectively protect tomato crops. *Pseudomonas and Bacillus* are considered to be the most interesting PGPR species for the biological control of *Cmm* worldwide. Improving plants’ innate defense mechanisms is one of the main biocontrol mechanisms of PGPR to manage bacterial canker and to limit its occurrence and gravity. Herein, we further discuss elicitors as a new management strategy to control *Cmm*, which are found to be highly effective in stimulating the plant immune system, decreasing disease severity, and minimizing pesticide use.

## 1. Introduction

The world’s population is steadily increasing and may exceed over 9 billion people by 2050 [1]. However, approximately 2 billion people worldwide are moderately to severely food insecure and face an increased risk of hunger, malnutrition, and health disorders. Therefore, agricultural systems are challenged with finding appropriate solutions for more sustainable food production, with the requirement to increase total food production by 70–100% to satisfy the global population’s needs and meet the growing consumer demand for healthy food that is free of synthetic agrochemicals [2]. Plant pathogens constitute a severe challenge to agricultural productivity and food production worldwide, with spillover effects on natural resources [3]. Every year, crops are damaged by plant diseases caused by phytopathogens, which affect 10 to 20% of the world’s production, resulting in significant yield losses estimated at billions of dollars and preventing 800 million people from being adequately fed [4]. Among these pathogens, there are over 200 species of phytobacteria which could cause serious diseases in agricultural ecosystems worldwide. Indeed, most research on the interaction of bacterial pathogens with their target plants has focused primarily on the Gram-negative group, as they are the major soil pathogens and are readily available for molecular analysis. In contrast, the Gram-positive group of phytobacteria has unfortunately not attracted the same interest from molecular phytopathologists, even though some of them have caused significant crop losses in agriculture [5].

The Gram-positive *Clavibacter michiganensis* subsp. *michiganensis* (*Cmm*) is the causal agent of bacterial wilt and canker of tomato plants [6], which is considered one of the most potentially contagious and destructive diseases of this crop [5,7]. This bacterium is a seed-borne pathogen that is widespread in different areas of tomato production around the world, and has caused devastating epidemics, thus resulting in significant financial losses in greenhouse and field production [8,9]. It has become an economically serious threat to tomato producers worldwide [10]. For this reason, the European and Mediterranean Plant Protection Organization (EPPO) has declared *C. michiganensis* subsp. *michiganensis* as a quarantine organism at the international level [11].

Despite the seriousness of tomato bacterial canker, no control strategy has proven to be completely effective to date [12]. Since no *Cmm*-resistant seeds are commercially available, and genetic progress in selecting resistant lines remains modest, the management of bacterial canker remains difficult [13]. Currently, *Cmm* has been controlled primarily by strict prevention, which is aimed at reducing the risk of *Cmm*-spread and new epidemics [14]. The most frequent methods used to control *Cmm* infection include the use of various synthetic pesticides and antibiotics [15]. However, the widespread application of these agrochemicals has led to growing concerns about environmental pollution and health risks [16]. In addition, chemical control is costly and ineffective in managing bacterial canker [14]. Therefore, researchers have focused on developing more efficient and safer alternatives to manage the disease, while improving tomato crop quality and production. This has become a major priority in modern agriculture [17].

The use of PGPRs (plant growth-promoting rhizobacteria) as biocontrol agents (BCAs) has emerged as a promising alternative to synthetic chemicals, providing a cost-efficient and ecological approach to plant preservation [18,19]. PGPRs are free-living bacteria in the soil that have the potential to promote plant growth, improve crop yield, and limit pathogen infection through complex direct or indirect mechanisms, including growth promotion, antibiosis, and induced resistance in host plants, thereby contributing to effective disease control [20,21]. Therefore, PGPRs represent powerful sustainable agriculture tools which are now a common practice worldwide and a key trend for the future [22].

This review article illustrates the progress made on tomato bacterial canker and disease management strategies adopted to control *Cmm*, focusing first on the multiple pathways of action of BCAs used to successfully protect tomato crops, bringing together recent research results. We will then discuss elicitors used to boost the plant immune system to reduce disease severity.

**Figure 1:**
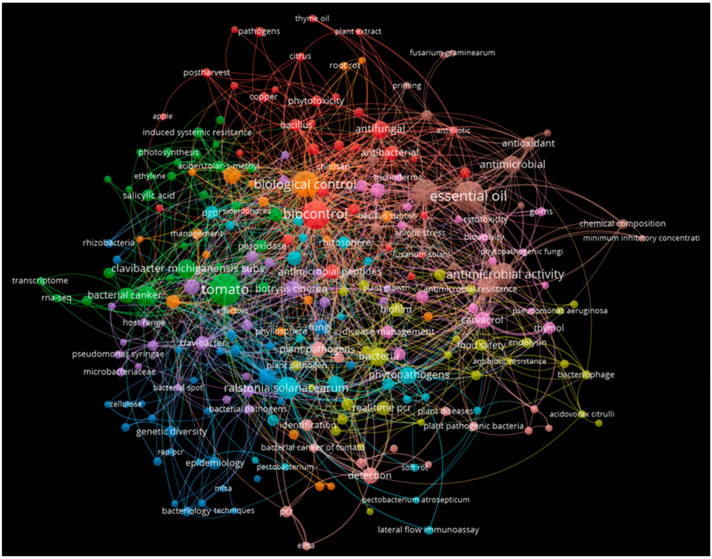
The bibliometric analysis illustrates the distribution of the most relevant articles in the control of bacterial canker of tomato caused by the pathogenic actinobacterium *Clavibacter michiganensis*.

In this review, using the SCOPUS database [23], bibliometric data were extracted using the specific research keywords “*Clavibacter michiganensis*” and “control”. The bibliometric analysis was constructed using the VOSviewer processing software (v1.6.9., Leiden University, Leiden, The Netherlands). The analysis shows the distribution of the most relevant articles in the control of bacterial canker of tomato caused by the pathogenic bacterium *Cmm*. The network analysis indicates the correlation between the keywords found and the overall perspective of current research in this area (Figure 1).

## 2. Overview of *Clavibacter* Sequenced Genomes

**Figure 2:**
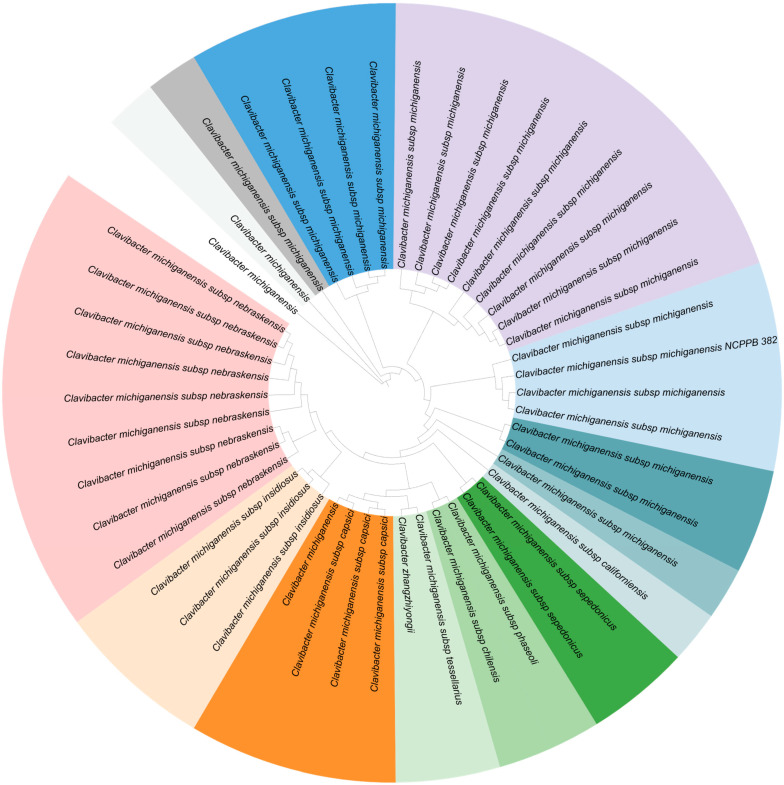
A genome-based phylogenetic tree based on 46 complete genome sequences of the genus *Clavibacter*. The tree was constructed with the Bacterial and Viral Bioinformatics Resource Center (BV-BRC) [24]. Only Clavibacter strains that have chromosome-level genome assembly were selected for analysis. The tree was visualized by using ITOL [25].

Forty-six complete genomes of *Clavibacter* strains were extracted at the Bacterial and Viral Bioinformatics Resource Center (BV-BRC) [24]. To evaluate the relevancy of the sequenced strains among the *Clavibacter* genus, a genome-based phylogenetic tree based on 46 complete genome sequences was constructed. The tree was built with the Bacterial and Viral Bioinformatics Resource Center (BV-BRC) [24]. Only *Clavibacter* strains that have chromosome-level genome assembly were selected for analysis. The tree was visualized by using ITOL [25] (Figure 2).

All strains have their genome split into one chromosome and up to three plasmids. The whole genome sizes span from 3 to 3.4 Mb, and the GC content is about 72%. The genome characteristics as well as the project information of different genomes of *Clavibacter* are presented in Table S1.

## 3. Importance of Bacterial Canker Disease

Tomato (*Lycopersicon esculentum* Mill.) is widely consumed and recognized as one of the world’s most widespread vegetable crops [26]. It accounts for 72% of the global value of fresh vegetables produced [27], achieving a global production of 182 million tons in 2018 [28]. In Morocco, tomato cultivation holds a prominent place in the export-oriented agricultural economy [29]. The crops are mainly grown on 15,239 hectares in the Souss-Massa region, with a total production of 1,231,250 tons and productivity of 81 tons/hectare in 2018 [28]. Furthermore, tomato is an indispensable part of the human diet, and its production provides income to many smallholder farmers in the poorest regions of the globe [30,31]. However, it is vulnerable to a broad variety of diseases, which significantly impact plant growth and even survival, thereby affecting crop quality and production [18]. Bacterial canker caused by the actinobacterium, *C. michiganensis* subsp. *michiganensis* is among the most devastating and contagious diseases that severely affect tomato plants [5,6,12]. It was first identified by Smith in Michigan, USA in 1909 [32]. The *Cmm* is a seed-borne pathogen [33]. Its long-distance movement is mediated by infected seeds, which may explain its current dispersion in most tomato-producing areas around the world [34,35,36,37]. *C. michiganensis* has caused devastating epidemics, resulting in severe economic damage in greenhouses and open-field production [12,38]. In addition, this destructive disease has severely affected the production and performance of tomato crops, with substantial yield losses ranging from 20 to 84%, thereby posing a real economic threat for tomato growers worldwide [10]. For this reason, the EPPO has declared *C. michiganensis* subsp. *michiganensis* as a quarantine organism at the international level [11]. Indeed, the severity of bacterial canker depends on several factors including the year, cultivar, cultural practices, phenological stage of host infection, weather conditions, and inoculum concentration [8,12].

The bacterial canker has been identified in Morocco since 1942 and has drastically damaged all tomato cultivation areas, with varying severity in different regions. In particular, the bacterial pathogen *Cmm* has been the principal reason for the premature death of tomatoes in the Souss-Massa-Draa valley in the Agadir region, affecting the yield and causing a severe decrease in fruit weight under field conditions, which represents a serious threat to tomato cultivars [29,39,40].

## 4. How Does *C. michiganensis* subsp. *michiganensis* Attack Tomato Plants?

**Figure 3:**
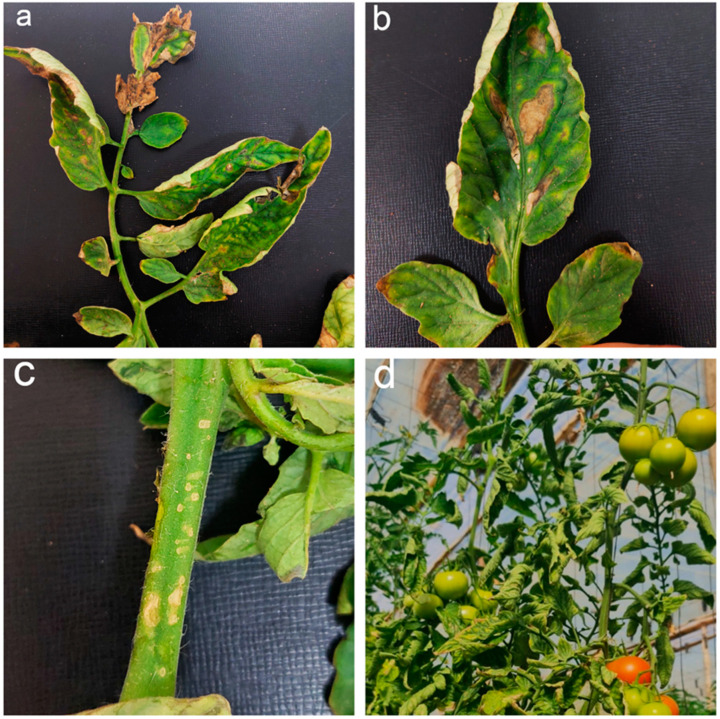
Typical bacterial canker symptoms on tomato plants; (**a**) marginal necrosis of leaflets and petioles, (**b**) areas of the desiccated leaf, (**c**) small white blister like lesions in the stem, and (**d**) unilateral wilting of leaves of tomato plants.

Tomato plants affected with *C. michiganensis* exhibit a variety of symptoms depending on several factors, such as the type of infection, the host’s age at the time of infection, cultivar susceptibility, virulence, and tomato growing conditions (temperature-humidity) [12,33]. In a systemic infection, the pathogenic bacterium typically invades plant vascular tissues through newly opened wounds on the surface of roots, stems, and leaves. In this case, plants are infected as seeds or young seedlings in the early phases of growth [7,8,14]. After penetration, *Cmm* ends up in the xylem vessels, where it has the potential to multiply rapidly and proliferate to high densities of $10^{4}$ to $10^{8}$ CFU per mg of tissue, provoking a decline in the hydraulic conductivity of the stem and leading to the unilateral wilting of leaves and leaflets (Figure 3) and the subsequent development of necrosis and cankers on the stems and petioles (Figure 3), vascular discoloration with brown streaks, and the wilting and ultimate death of the plant [12,41]. Typically, infected plants take up to 80 days to develop systemic symptoms under optimal environmental conditions, including temperature (25–28 °C), and high humidity [42]. In that case, the pathogen attacks the target plant at the late stages of its development via natural inlets such as stomata, hydathodes, or broken trichomes [33,43]. Consequently, a localized infection occurs on mature tomato plants, causing marginal necrosis of leaflets, the most common trait observed during field outbreaks and usually visible within 3–5 days [8]. Later, necrotic bird’s eye spots developed, surrounded by white halos on tomato fruits, and small blister-like lesions on leaves or stems (Figure 3) [8,12,44]. These plants could be asymptomatic and harbor latent infections, providing the principal sources of contaminated seeds and serving as infection sources during the next growing season [12,14].

## 5. Characteristics and Transmission Modes of *C. michiganensis* subsp. *michiganensis*

*C.michiganensis* was initially considered a phloem parasite but later was detected as a bacterium infecting xylem tissue and tomato fruit. It is an aerobic, non-motile, and non-sporing actinomycete that can develop at temperatures of 20 to 30 °C, with optimal growth at 25 °C [14]. *Clavibacter* grows in the xylem of plants at a pH of 5, while its ideal pH for development is between 7 and 8 [10,14].

The molecular mode of *Cmm* infection is severely complicated and poorly understood [5,45], because the bacterium acts as an endophyte during the first stages of infection, and thereafter, *Cmm* reverses its behavior by causing disease symptoms under certain favorable conditions [14]. Therefore, researchers have extensively identified the molecular determinants implicated in *clavibacter* pathogenicity [8]. Interestingly, the publication of the genome sequences of the wild-type strain NCPPB382 has offered an important platform for genetic research into host-pathogen interactions, as well as information on disease induction processes [8,14,45]. The reference strain, *Cmm*382, is characterized by a significant GC content in its genome [45]. It harbors a circular chromosome and two circular conjugative plasmids, pCM1 and pCM2, which are key contributing factors to pathogenicity [46,47]. Each of these contains a major virulence gene that is essential for systemic infection and the complete development of wilt in affected tomato plants [45]. pCM1 (27.4 Kb) harbors celA, encoding endo-β-1,4 glucanase, while pCM2 (70 Kb) carries Pat-1, encoding a putative serine protease [48,49]. Additionally, *Cmm*382 can secrete a wide variety of active enzymes involved in plant cell wall deterioration, such as pectate lyases, xylanases, and cellulases, promoting bacterial invasion and nutrient procurement [10,45]. Moreover, *Cmm*382 harbors a chromosomal pathogenicity island (PAI) with low G + C content (65%), and is defined as chp/tomA. The PAI may cluster in two subregions: the chp subregion, which carries many genes encoding putative proteases, and the tomA subregion, which carries tomA and encodes tomatinase [45,48,50]. The latter is responsible for tomatin degradation and supplies a basal defense to tomato plants. It carries all the genes essential for invasion, efficient colonization, and the ability to evade or remove plant responses [8]. In fact, the removal of pCM1 or pCM2 reduces the pathogenicity, whereas curing both plasmids produces a non-virulent strain (strain *Cmm*100) that can still grow as an endophyte like the wild type [7].

This bacterial pathogen may survive in infested seeds and plant debris for varying lengths of the period, and it can persist in the soil for up to four years [10,39]. In addition, it has the ability to survive epiphytically in alternative hosts and volunteer plants [8,14]. These are all frequent sources of *Cmm* primary inoculum [12,39]. Infected seeds are the main vector for *Cmm* transmission and long-distance spread, allowing its introduction into areas that were previously free of the disease [8,42]. Rates of the pathogen spread from seeds to plants can vary from 0.25 to 85% [14]. A severe epidemic can be initiated with a transmission rate of only 0.01% [14,39]. Secondary transmission can occur when *Cmm*-infected plants contaminate neighboring healthy plants, which is promoted primarily by various agricultural operations, including transplanting, pruning, and harvesting. In addition, *Cmm* can be dispersed by rain splash, overhead irrigation, or chemical spraying during routine practices in nurseries and greenhouses [8,10].

## 6. Disease Management Strategies

Despite significant efforts by researchers to find appropriate methods to control bacterial canker, disease management remains a critical challenge for tomato production worldwide [16]. This could be due to the sporadic nature of bacterial canker, which makes its management extremely complicated once it is triggered. [14] Unfortunately, to date, no control method has proven to be completely effective [51], as there are no commercially available *Cmm*-resistant cultivars yet, as well as because of the pathogen’s genetic diversity and genomic heterogeneity [36]. In addition, research investigating the chemical control of *Cmm* is scarce and has shown variable results] [36,52]. The main agrochemicals employed are copper-containing compounds, including copper sulfate, copper hydroxide, and antibacterial compounds such as mancozeb, streptomycin, and their combinations [10,36]. However, none of these treatments are credible and consistent in controlling the bacterium when conditions favor the canker development, allowing only the reduction in the pathogen population’s surface area [14]. Moreover, their use is not encouraged because they have a major negative impact, leading to increasing concern about environmental pollution and ecological disruption, in addition to human health risks and toxic effects on beneficial organisms [16]. Hence, the most obvious measure to effectively control this destructive plant pathogen and minimize the substantial crop losses caused by *Cmm* is prevention, relying principally on the use of pathogen-free seeds and transplants, and on strict sanitary measures imposed by European Phytosanitary Legalization such as the removal of plant debris, clean transportation practices, crop rotations, adequate hygiene in greenhouses, and the disinfection of planting equipment and materials [12,14,53]. Similarly, the Good Seed and Plant Practices (GSPP) organization encourages the marketing of pathogen-free seeds [5]. In parallel, the implementation of advanced diagnostic procedures is an essential step to reduce the risk of disease spread while preventing the occurrence of new outbreaks [36]. Finally, it is necessary to identify safer and more effective alternatives that will increase crop quality and production and effectively control this disease.

## 7. The Use of PGPRs as an Alternative Biocontrol Strategy

**Figure 4:**
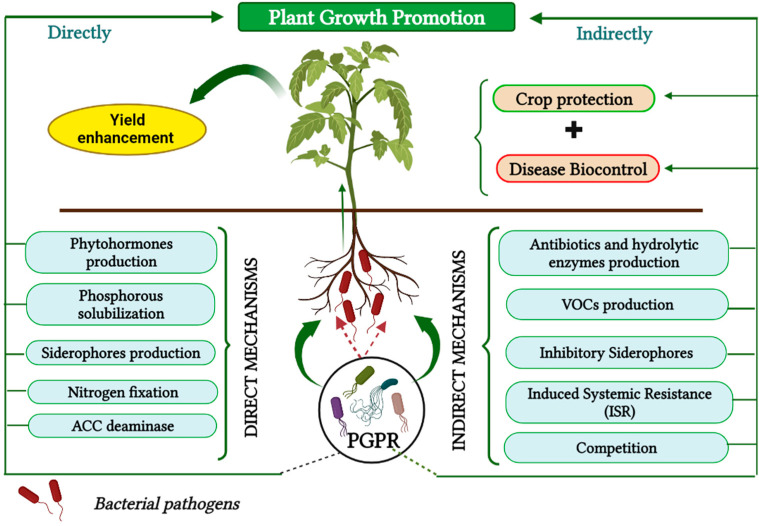
Schematic illustration of direct/indirect mechanisms of PGPRs to promote plant growth, maintain crop protection from pathogens, and improve yield.

To prevent plant diseases, multiple biological strategies have been adopted, avoiding the intensive use of synthetic agrochemicals in agronomic vegetable production [16]. The use of plant growth-promoting rhizobacteria (PGPR) as biocontrol agents has been recommended as an ecological and economical approach to disease management and a promising antibacterial alternative to agrochemical methods, while conserving natural resources [21,54]. As part of sustainable agriculture, PGPRs have become a common practice worldwide, increasing biodiversity, improving crop yields, and limiting pathogen infection [19]. PGPRs represent an important group of free-living soil bacteria that can effectively colonize plant roots [17], and some of these motile rhizobacteria can develop an endophytic bacterial population, reflecting their adaptability to specific ecological niches [55]. They are well-known for optimizing the development and performance of plants by affecting plant growth through direct or indirect processes [17] (Figure 4). Direct mechanisms adopted by PGPRs include nitrogen fixation, phosphate solubilization, the synthesis of growth regulators (phytohormones), and the induction of ACC deaminase, in addition to the biosynthesis of siderophores [56,57].

PGPRs may indirectly promote plant growth by preventing deleterious soil pathogens, or at least reduce their ability to induce diseases, either through the secretion of secondary metabolites, including antibiotics, hydrolytic enzymes, and volatile organic acids, or by competing with pathogens for ecological niches or nutrients (Carbon/Energy Sources) [17,21,55,58]. This is done by inducing systemic resistance (ISR) in host plants, which represents a physiological enhancement of the plant’s resistance capacity (Figure 4), leading to the strengthening of its innate response mechanisms and contributing effectively to the biocontrol of pathogens, thus limiting the occurrence and severity of diseases [21,59]. These valuable characteristics of PGPRs can be used to improve food safety and facilitate their emergence in various applications, especially in biotechnology [56,60].

### 7.1. Mechanisms of PGPRs to Control Bacterial Canker

*Pseudomonas* spp. is regarded as one of the widespread and well-studied genera of beneficial rhizobacteria within the diverse bacterial communities of the rhizosphere [17,61]. They are Gram-negative, aerobic, mobile, and ubiquitous bacteria, characterized by their ability to produce a biofilm, which allows them to attach to the surface of plant roots to easily exercise their beneficial mechanisms on the host plant [62,63], and even enable them to effectively adapt to environmental stresses, especially by actively suppressing many phytopathogenic bacteria [21,64,65]. Regarding the bacterial canker of tomato, many rhizobacteria with antagonistic properties against *C. michiganensis* have been identified and analyzed [64,66,67,68,69] (Table 1 and Table 2). However, fluorescent *Pseudomonas* have been reported as the most powerful strains belonging to *Pseudomonas* in the biocontrol approach [21,70]. For this fact, exogenous treatments of roots and seeds with *P. fluorescens* strains were performed before transplanting to prevent the occurrence of bacterial canker in the greenhouses, as the research of Amkraz et al. (2010) approved the efficacy of fluorescent *Pseudomonas* isolates in decreasing the severity of canker on tomatoes, with rates of reduction of disease incidence ranging from 61 to 83.19%. Therefore, these strains have received increasing attention as a valuable source of biological control of tomato diseases around the world [19].

#### 7.1.1. Direct Mechanisms

Along with *Pseudomonas, Bacillus* spp. are the most frequent bacteria found in the rhizosphere [21]. For a long time, these bacteria have been acknowledged as influential agents in plant growth, and have been viewed as a promising approach to enhance plant productivity and yield. This is due to their capacity to generate various biologically active compounds (listed in Table 2), some of which are particularly noteworthy, such as the plant hormones gibberellins and cytokinins, as well as their notable production of indole-3-acetic acid (IAA) [19,70]. All of these compounds can effectively increase plant nutrient availability and improve root parameters [21,71]. Therefore, seed treatments with both *Pseudomonas* and *Bacillus* strains have been used as biofertilizers, because it has been proven that they can significantly improve the performance and quality of tomato plants, leading to high yield increases in field experiments [15]. Similarly, recent research conducted by Escamilla-Silva & Luz (2021) revealed that treatment with *Bacillus. cereus* strains resulted in the highest plants heights and showed a considerable increase in the fresh and dry weight of their roots and shoots, this being associated with the ability of *B. cereus* to generate gibberellins, in particular, gibberellic acid (GA3) [72]. In addition, *Bacillus. amyloliquefaciens* has been shown to produce significant levels of IAA, a key phytohormone that increases the tomato plant’s potential to absorb water and nutrients, regulates plant growth, and participates in the implementation of its immune defense responses [67,73]. However, research revealed that *Pseudomonas* strains can generate IAA at greater levels than others, for example, IAA was produced by *Pseudomonas. aeruginosa* FG106 isolates, reaching a maximum production of 211 µg/mL, resulting in increased root length [74]. Furthermore, numerous fluorescent *Pseudomonas* species are potent phosphate solubilizers, including *P. aeruginosa*, *P. entomophila* 23S, and *P. fluorescens* [67,74,75] (Table 1), through which they can promote tomato plants growth, knowing that phosphorus is an extremely important micronutrient for organic crop development [19], and some of these pseudomonas strains can successfully synthesize ammonia, which enhances the host plant’s uptake of nitrate and ammonium [74].

#### 7.1.2. Indirect Mechanisms

PGPRs might potentially be powerful biocontrol agents, suppressing harmful pathogens, and thus indirectly stimulating plant growth [17] (Figure 4). Indeed, numerous *Pseudomonas* strains are known for their direct antibiosis against *clavibacter michiganensis*, which is one of the most potent and successful biocontrol functions (Table 1), relying on the secretion of a diverse spectrum of secondary metabolites with antimicrobial properties, such as phenazines, including phenazine-1-carboxylic acid (PCA), phenazine-1-carboxamide (PCN), as well as pyocyanin (PYO), pyrrolnitrin, and/or pyoluteorin, 2,4 diacetylphloroglucinol (DAPG), hydrogen cyanide (HCN), siderophores, volatile organic compounds (VOCs), and various enzymes [63,70,76].

1. Antibiotics and VOCs production

Several studies have shown that the presence of *Cmm* allows *P. brassicacearum* LBUM30 to actively synthesize DAPG and HCN through the increased expression of *phlD* and *hcnC* genes, respectively. Therefore, it helps in limiting the *Cmm* growth in vitro and reduces the occurrence of bacterial canker under *planta* circumstances [64,66]. Indeed, DAPG is one of the most effective and well-studied antibiotics. Its production was found to be associated with an improved capacity of *P. brassicacearum* to colonize the rhizosphere of tomato plants, thus resulting in greater biofilm formation [66]. HCN suppresses the growth and metabolism of *Cmm*, thereby delaying disease development and protecting tomato plants from damage [51]. Deng et al. [69] showed that *P. chlororaphis* UFB2 exhibited effective biocontrol activity against *C. michiganensis* because its genome sequencing has shown that the UFB2 strain harbored genetic islands encoding various secondary metabolites with an antibiotic function [69]. Moreover, research by Raio et al. [68] proved that the antimicrobial potency of *P. chlororaphis* M71 depends primarily on the generation of phenazines, of which the antibiotic PCA exhibits high redox activity [77]. Furthermore, various VOCs could be generated by *P. chlororaphis* strains such as dimethyl disulfide and methanethiol, which were potentially able to suppress the *Cmm* growth in vitro. The presence of these characteristics suggests that P. chlororaphis could have a broad application in the biocontrol of bacterial canker [68].

2. Inhibitory siderophores production

Under iron deficiency conditions, various PGPR species can produce siderophores, which are small molecules with an iron uptake system capable of chelating Fe^3+^ molecules with high specific activity [21]. Indeed, siderophores have been widely implicated in biocontrol activity as virulence factors, and represent a primary function in limiting the iron source required for the growth of pathogenic bacteria, so they are an essential asset of PGPRs to survive and thrive in a complex ecosystem [17,19]. Recently, the investigation by Abo-Elyousr et al. [67] showed that isolates of *B. subtilis, B. amyloliquefaciens*, and *P. fluorescens* can generate significant levels of siderophores. Therefore, this leads directly to the production of other antimicrobial substances by promoting the supply of iron minerals to rhizobacteria, which in turn would act as antagonists towards the pathogenic bacterium via functioning as stressors in the initiation of host resistance [67,78].

3. Lytic enzymes production

PGPRs can produce a variety of cell wall degrading enzymes, and hydrolytic enzymes that suppress the pathogen through cell lysis and parasitism [58,79]. As well, recent research by Oloyede et al. [51] reported that the non-indigenous strains *Alcaligenes faecalis* and *Acinetobacter* sp. were very effective in decreasing the severity of bacterial canker, as these strains could attack the bacterial cell wall by secreting a variety of enabling enzymes such as cellulase, protease, pectinase, and β-1,3-glucanase. *Pseudomonas* were also found to produce lytic enzymes, primarily by *P. aeruginosa* FG106 and *P. chlororaphis* M71, and thus contributed to disease suppression [68,74].

4. Lipopeptides surfactants production

The use of *Bacillus* species with antagonistic properties is expanding rapidly, as many studies have reported that they can secrete a diverse array of secondary metabolites (Table 2). As an example, *C. michiganensis* growth was shown to be inhibited in vitro and in vivo by the *B. subtilis* strain DJM-51 through its butanol-extracted compounds and its culture supernatant [80]. Furthermore, *Bacillus* species can synthesize a broad spectrum of lipopeptides surfactants, including iturins, surfactins, and mycosubtilin [81]. Grady et al.’s [82] research focused on surfactins generated by *Bacillus velezensis* 9D-6, which were isolated and tested on agar plate assays to screen their antimicrobial activities. The surfactin [Leu7] C14 (surfactin B) and surfactin [Leu7] C15 (surfactin C) were found to be effective inhibitors of *C. michiganensis*. Similarly, Laird et al. [83] approved the efficacy of the microbial antagonism of *B. velezensis* 1B-23 and *Bacillus* sp. 1D-12, revealing that they could secrete surfactin [Leu7] C13 (surfactin A), in addition to surfactin B and surfactin C. All of these substances served as potent antibiotics specifically directed to suppress the growth of *C. michiganensis* in vitro, through the disruption of bacterial membranes, and also contributed to the reduction of disease symptoms in vivo. Therefore, it was indicated that *B. velezensis* could be developed as a biopesticide for sustainable agriculture [82,83].

**Table 1:** Overview of the modes of action of *Pseudomonas* strains to enhance plant growth and to control *C. michiganensis*.

Biocontrol Agent	Bacterial Traits		References
***Pseudomonas fluorescens***	**Siderophores production**	Pyoverdine (pseudobactin) Pyochelin	[67,84]
	**Antimicrobial compounds**	HCN Phenazines (PCA)	[67,85] [86]
	**Growth promoting factors**	IAA Phosphate solubilization	[85] [67]
***Pseudomonas Brassicacearum* LBUM300-LBUM323**	**Siderophores production**		[64]
	**Antimicrobial compounds**	DAPG HCN PCA	[66] [64]
	**Growth promoting factors**	Biofilm formation	[66]
***Pseudomonas aeruginosa*** ***FG106-BRp3***	**Siderophores production**	Pyoverdine Pyochelin	[87] [59]
	**Antimicrobial compounds**	HCN Phenazines: (PCA-PCN-PYO) Pyoluteorin	[74] [88]
	**Biosurfactant**	Rhamnolipids	[74]
	**Growth promoting factors**	IAA Phosphate Solubilization Potassium Solubilization Ammonium production ACC deaminase activity Biofilm formation	[74] [88]
	**Enzymes**	Proteases (Elastase, Alkaline protease) Chitinase	[74]
***Pseudomonas chlororaphis M71/UFB2***	**Siderophores production**		[68]
	**Antibiotic compounds**	DAPG HCN Phenazines (PCA)	[69] [77] [68]
	**VOCs**	Methanethiol Dimethyl disulfide	[68]
	**Hydrolytic enzymes**	Protease Lipase	
	**Growth promoting factors**	Biofilm production	
	**Production of AHLs**		
***Pseudomonas entomophila 23 S***	**Siderophores production**		[75]
	**Antibiotic compounds**	HCN	
	**Growth promoting factors**	IAA Phosphate Solubilization	

**Table 2:** Overview of the modes of action of *Bacillus* strains to foster plant growth and to control *C. michiganensis*.

Biocontrol Agent	Bacterial Traits		References
***Bacillus subtilis***	**Siderophores production**		[67]
	**Antimicrobial compounds**	HCN Surfactin Butanol	[80] [67]
	**Growth promoting factors**	IAA	[89]
***Bacillus amyloliquefaciens***	**Siderophores production**		[90]
	**Growth promoting factors**	IAA Phosphate solubilization Growth in nitrogen-free medium	[67]
	**Enzymes production**	Cellulase Chitinase Lipase Protease	[90]
***Bacillus cereus***	**Growth promoting factors**	Gibberellic Acid (GA3) Phosphate solubilization	[72]
***Bacillus velezensis 9D-6*** ***Bacillus sp. 1D-12***	**Antibiotic compounds**	Surfactin A Surfactin B/C	[82,83]

5. Induced plant defense responses

**Figure 5:**
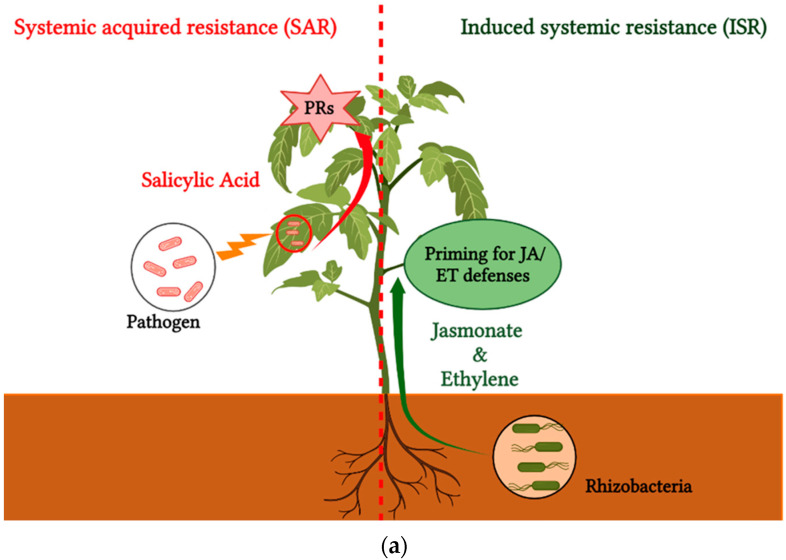

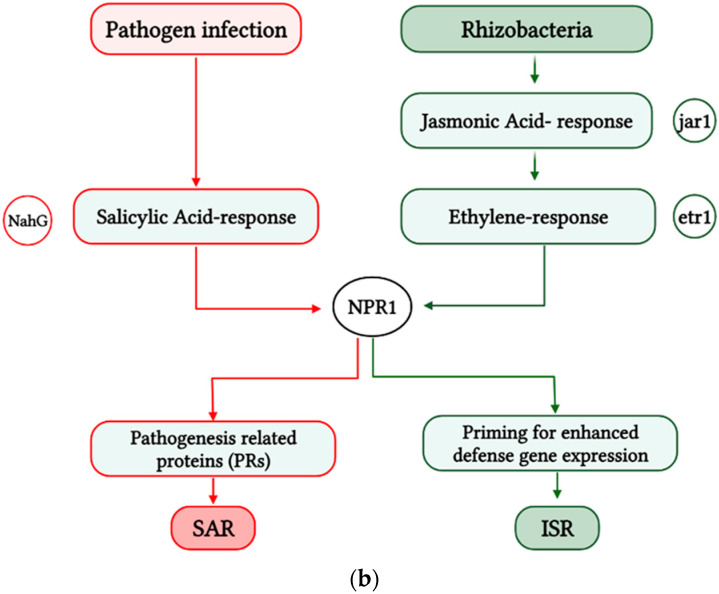
Schematic illustration of induced immune responses: (**a**) an image represents systemic acquired resistance (SAR), which is triggered after local infection of plants by phytopathogens. SAR provides resistance to biotic and abiotic stresses; whereas induced systemic resistance (ISR) is activated by plant growth-promoting rhizobacteria (PGPR) and confers resistance to biotic stresses. (**b**) a flowchart shows the signal transduction pathway of SAR, which relies on the salicylic acid (SA) pathway, encoded by NahG, while ISR involves jasmonic acid and Ethylene, encoded jar1, and etr1, respectively.

All over the world, plants are constantly attacked by various harmful agents, which increase the generation of reactive oxygen species (ROS), thus causing oxidative stress (OS), which damages the function of nucleic acids, chloroplast polar membrane lipids, and allows the inactivation of enzyme systems [91,92]. Thus, plants respond immediately to attackers by triggering a series of defensive mechanisms, presenting a set of structural barriers, and producing inhibitory metabolites to block or mitigate pathogen infection [93]. If the pathogen successfully persists and surmounts the hypersensitive plant responses, it must still defy the well-structured plant defense responses, which include systemic acquired resistance (SAR) [94]. The SAR represents a particular type of induced resistance in plants, and is defined by a specific signaling pathway, namely salicylic acid (SA), which is released systemically after a localized pathogen attack, and generally leads to the expression of the pathogenesis-related (*PR*) genes (Figure 5) [95,96,97]. These PR proteins include a range of enzymes such as β (1–3) glucanases, and chitinases, which have an inhibitory action that can directly lyse invading cells and attack pathogenic structures [17].

Beneficial microbes, especially PGPRs, are typically associated with induced systemic resistance, which is initiated by jasmonic acid (JA) and ethylene (ET) [95,98]. These phytohormones are key players that act as signaling molecules in the implementation of a series of defense related-genes, regulating SA-independent systemic immunity [99], Therefore, this helps to enhance host plant defensive responses [91,100], and through which PGPRs effectively defend crops against phytopathogens [101]. Indeed, PGPR- mediated ISR is generally based on priming as an important process, allowing cellular defenses to be activated more rapidly and more strongly, leading to very high levels of resistance against many types of plant pathogens [17,102], including *Clavibacter michiganensis* (Table 3). As reported by Takishita et al. [75], who demonstrated that *Pseudomonas sp. 23S*, along with its direct inhibitory effect on *Cmm* (siderophore production), is actively involved in the stimulation of defense mechanisms on tomato plants, which implicates SA in its signaling pathway instead of JA and ET, the application of *Pseudomonas sp. 23S* significantly increased the transcript level of the *PR1a* gene that encodes a pathogenesis-related protein, and is also used as a marker for SA [103]. The biotrophic nature of *Cmm* makes it an ideal target for SA-dependent ISR [5,75]. Furthermore, pre-treated tomato plants were shown to have a higher and faster response capacity than untreated plants [75]. Aksoy et al. [104] proved that inoculation with *P. putida* CKPp9 can initiate systemic resistance, allowing the plant to synthesize large amounts of phenolic compounds in tomato leaves, particularly catechins and chlorogenic acid. Catechin accumulation was highest in CKPp9+*Cmm* treated tomato plants, which was almost 10-fold higher than in other treatments, confirming that there is a close relationship between the overproduction of catechin and the reduction of disease severity. Similarly, better results were found for pretreatment with *A. chroococcum,* which was associated with the highest accumulation of phenols, reaching up to 54.1 mg/g, and provided significant levels of flavonoids relative to control plants [105]. Interestingly, phenolic compounds are natural constituents of plants that are known to exert crucial control over metabolic processes, lignin biosynthesis, and phytoalexin accumulation. Therefore, this will improve the efficiency of the plant’s defense systems against pathogen attacks [106,107]. Moreover, the study conducted by Kolomiiets et al. [105] showed that the leaves of treated plants had very high chlorophyll and carotenoid content compared to untreated control plants; this increase reflects the proper functioning of the photosynthetic process and the development of the bacterial canker resistance mechanisms. Furthermore, pretreatment with *B. subtilis* conferred high resistance to tomato plants through increased peroxidase activity. A recent study by Escamilla-Silva et al. [72] indicated that *B. cereus* inoculation strongly stimulates the innate defense system in tomato plants, causing an increase in gene expression of the enzymes Phenylalanine ammonia-lyase (PAL) and chalcone synthase (*chs*), both of which lead to the biosynthesis of phenylpropanoids (flavonoids). Indeed, these compounds have been involved in crop preservation in multiple models of plant-microbe interaction.

## 8. The Use of Elicitors as a New Target in Agriculture

### 8.1. Salicylic Acid (SA)

**Figure 6:**
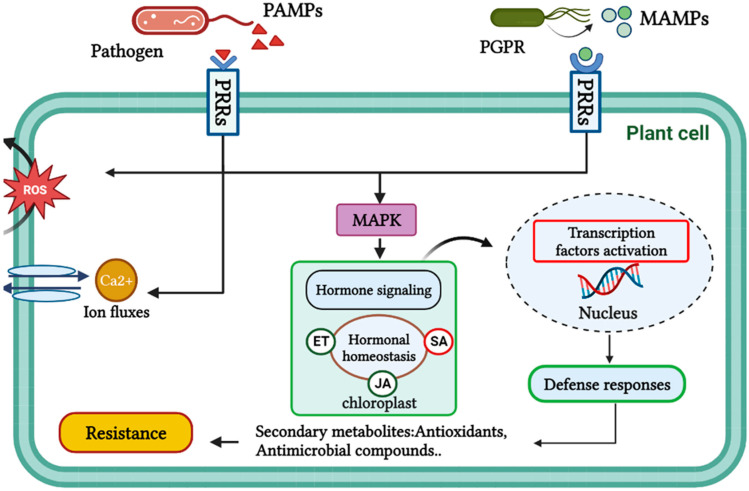
Schematic representation showing the activation processes of plant defense responses. These defenses are triggered when a stimulus is perceived by PRRs located on the surface of plant cells. Pathogens and PGPR are both capable of inducing the production of molecular signals that activate hormonal signaling pathways in plants. Specifically, ROS, Calcium, and MAPK are among the key molecular signals that are increased in response to these microorganisms, ultimately leading to the development of plant resistance.

Plants possess an innate defense system that effectively detects and provides an appropriate response to pathogen attacks; the first line of defense immunity is potentially based on the perception of the pathogen as molecular patterns (PAMPs) through pattern recognition receptors (PRRs) located on the plant surface, thus inducing plant immunity, which is called pattern-triggered immunity (PTI) (Figure 6) [91]. Many studies have shown that *Clavibacter michiganensis* infection can stimulate fundamental defense responses in tomato plants, resulting in higher transcript levels and the stronger expression of the most defense-related genes [108,109]. About 7% of tomato genes were found to respond significantly to *Cmm* infection, especially pathogenesis-related genes, which were strongly up-regulated [110]. According to a Gene Ontology enrichment analysis, all overrepresented genes are generally associated with defense signal transduction, such as plant hormone signals, redox regulation, calcium signaling, and increased protein turnover [108]. It was found that the amounts of SA were significantly elevated in *Cmm*-inoculated cotyledons, indicating that the host plant’s defensive mechanisms were greatly stimulated [110]. In this respect, Yokotani et al. [110] confirmed that the exogenous treatment of SA in tomato seedlings partially inhibited the bacterial proliferation of *Cmm* in tomato cotyledon, by triggering plant defense processes, especially through the expression of *WRKY* genes, which were strongly up-regulated and have the function of encoding transcription factors, and which may also serve to regulate the expression of defense-associated genes during tomato-*Cmm* interaction [111]. Overall, SA plays an important role and would be a beneficial technique for controlling *Cmm* in agriculture [110].

### 8.2. Beneficial Microbes

Various beneficial microbes are also recognized to activate plant immunity. Therefore, the term microbe-associated molecular patterns (MAMPs) has been used to classify conserved molecules, which are known to induce MAMP-triggered immunity (MTI) after their recognition by specific pattern recognition receptors (PRRs), resulting in the induction of signaling molecules such as (ROS), Ca^2+^, nitric oxide (NO), and the induction of antimicrobial compounds, in addition to the synthesis of pathogenesis-related proteins (PR), leading to increased plant resistance (Figure 6) [112,113]. Several examples of MAMPs have been cited, such as outer membrane lipopolysaccharides (LPS), basic elements of bacterial cell walls such as peptidoglycans, and glycoproteins, in addition to flagella [113,114,115,116]. Interestingly, some PGPRs can secrete a variety of molecules, termed elicitors, which are sensed by plant cells to trigger the ISR phenomenon, activate biochemical and physiological responses, and provide signaling functions in plants [101,117]. Many studies have investigated the various bacterial-secreted metabolites eliciting plant defense responses, including iron-regulated siderophores, antibiotics such as DAPG and pyocyanin, biosurfactants, and AHLs [116,118,119]. These molecules may contribute to SA accumulation and subsequently stimulate defense mechanisms such as phenolic biosynthesis, callose deposition, and stomatal closure [91]. Regarding tomato plants, a recent study by Jang et al. [120] demonstrated the ability of bioactive extracts from *Bacillus* strains H8-1 and K203 to increase the expression of the *PR-1a* gene, while decreasing the expression of the ethylene-related gene (*ACO*) was found to significantly reduce the occurrence and severity of bacterial canker under *planta* conditions. Therefore, this enhances plant defense responses through SA-dependent pathways and leads to the suppression of tomato wilt. Moreover, treatment with these extracts showed strong inhibitory activity against the *Cmm* viability and decreased the expression of its virulence genes, including *celA, celB -- pat1, and pelA1*, which respectively encode two major cell-degrading enzymes of *Cmm*: cellulase and pectate lyase, which are required to successfully infect and colonize tomato plants, and play a central role in attenuating its defense responses [5]. Therefore, bioactive extracts of H8-1 and K203 were effective in reducing the infection potential of *Cmm* by altering its virulence factors [120].

### 8.3. Fungal Elicitors

Several research studies have illustrated the efficacy of fungal biocontrol against a wide range of bacterial pathogens through several mechanisms, most notably fungal extracts and secretions, which can act as natural elicitors [121]. Indeed, the epiphytic nature of *Pseudozyma aphidis* and its dual mechanism of action, including antibiosis and induced resistance on tomato plants, have made this species one of the most effective in terms of the biological activity against *Clavibacter michiganensis* [122], and this is due to the secreted substance of *P. aphidis*, which were shown to inhibit *Cmm* in vitro as well as in planta. The occurrence and severity of bacterial wilt were greatly decreased when *P. aphidis* was applied to tomato plants before pathogen inoculation. Moreover, it was shown to be able to induce plant resistance by activating SA and ET- dependent resistance pathways, as revealed by the up-regulation of *PR1a* and *PTI5* marker genes. The research findings suggest that the plants- treated with this competent fungal agent were healthier compared to untreated plants [122].

### 8.4. Synthetic Elicitors

#### 8.4.1. Acibenzolar—S–Methyl (ASM)

In addition to biotic inducers, several compounds have been identified as synthetic defense elicitors. These are specific small molecules that are known to trigger plant defense responses, helping to defend against infections and reduce severe diseases without having direct antimicrobial effects, thus minimizing the negative effects of pesticides [123]. The benzothiadiazole derivative benzo-1,2,3- thiadiazole-7-carbothermic acid-S-methyl ester (acibenzolar-S methyl, ASM/BTH) has been widely used against *C. michiganensis* as a potent elicitor to stimulate the SAR signal transduction pathway and promote resistance in tomato plants, also reducing the disease severity [96,123,124]. Furthermore, several studies have examined the mechanisms implicated in the ASM-mediated resistance of tomato and its efficacy in reducing populations of *Cmm*, including research by Baysal et al. and Soylu et al. [96,125], who confirmed that tomato plants treated with ASM and then inoculated with *Cmm* showed enhanced expression of antioxidant enzymes, particularly peroxidase, PAL, and GPX (Glutathione-Peroxidase), which function as key protective enzymes for plant cells against oxidative stress damage. Interestingly, peroxidase is one of the main enzymes engaged in defensive mechanisms that include the lignification, the deposition of polyphenols, and cross-linking of cell wall proteins, which ultimately leads to the strengthening of the plant cell wall, so they can function as a mechanical boundary to resist pathogen penetration [97,126]. A correlation was also found between the application of ASM and the accumulation of chitinases, which act as lysozymes and may hydrolyze bacterial cell walls [125].

#### 8.4.2. INA and DPMP

Numerous studies have demonstrated the significant plant protection of 2,6-dichloro-isonicotinic acid (INA), which is considered to be one of the earliest synthetic elicitors discovered [123]. Similarly, various efforts have been made to identify other synthetic defense elicitors. For Example, 2,4-dichloro-6-{(E)-[(3-methoxyphenyl) imino] methyl} phenol (DPMP) has emerged as a new synthetic elicitor, known to induce a strong immune response even at very low concentrations, thus offering great potential for plant protection [127]. Recently, Bektas et al. [128] demonstrated that both elicitors, INA and DPMP, can induce defense mechanisms in tomato plants and successfully reduce the disease severity caused by *Clavibacter michiganensis,* without having direct toxic effects against *Cmm,* as the results revealed that the application of DPMP increased the transcript level of *PR-1*, while INA extensively increased *PR-5* gene expression. According to the results, these elicitors can significantly improve plant growth parameters and reverse the adverse effect of disease on plant performance.

## 9. Conclusions and Future Perspectives

Bacterial canker disease, caused by *C. michiganensis*, is becoming a major threat to tomato growers worldwide. Health and environmental concerns, as well as increasing consumer demand for pesticide-free food, have encouraged the use of PGPRs as a promising alternative to synthetic chemicals for sustainable agriculture. This review demonstrates the capacity of various PGPRs species to support plant growth and effectively protect tomato crops from *Cmm*-infection, as well as to improve yield and quality. This biocontrol process is owed to the existence of various characteristics of these rhizobacteria. We focused on presenting the PGPR modes of action and understanding positive interactions between tomato plants and PGPR strains to directly or indirectly enhance their growth. Improving plants’ innate defense mechanisms is one of the main biocontrol mechanisms of PGPR to manage bacterial canker, thereby limiting the disease occurrence and severity. In addition, we presented the different types of elicitors used to enhance the plant immune system and prevent devastating outbreaks in field production. Implementation of advanced diagnostic procedures is essential to prevent new outbreaks and reduce the risk of bacterial canker spread. Additional screening for more potent and possibly distinct PGPR species is desirable. A greater diversity of biocontrol agents will not only benefit basic research but may be necessary for sustainable agriculture. In addition, innovative methods for screening tomato plant immunity stimulants are desired to enrich the set of available compounds.

## Figures and Tables

**Table 3:** Mechanisms for stimulating induced systemic resistance (ISR) by biocontrol agents.

Biocontrol Agent	Compound/Gene	References
**Mechanism/activity**	Induced systemic resistance (ISR)	
***Pseudomonas 23 S***	Through SA in its signaling pathway Up-regulation of *PR-1a* gene Increased the transcript level of *ACO* (ET)	[75]
***Pseudomonas putida* CKPp9**	Increased biosynthesis of phenolic compounds in tomato leaves (catechin, chlorogenic acid)	[104]
***Azotobacter chroococcum***	Provided an accumulation of phenols and flavonoids in tomato leaves	[105]
***Bacillus subtilis***	Increased chlorophyll (a+b) and carotenoid content in tomato leaves Increment of the Peroxidase (POX) activity	[105]
***Bacillus cereus***	Increased total chlorophyll content in tomato plants. Increment of the activity of PAL and the expression of *chs* (contributed to flavonoids biosynthesis)	[72]

## Data Availability

The data presented in this study are available in the Supplementary Materials and via the accession numbers described in the Section 2 of this article.

## Supplementary Materials

The following supporting information can be downloaded at: https://www.mdpi.com/article/10.3390/microorganisms11030726/s1, Table S1: Project information and genomic features of genome sequences of different genomes of Clavibacter.
